# Primary aldosteronism: molecular medicine meets public health

**DOI:** 10.1038/s41581-023-00753-6

**Published:** 2023-08-23

**Authors:** Elena A. B. Azizan, William M. Drake, Morris J. Brown

**Affiliations:** 1 Department of Medicine, Faculty of Medicine, The National University of Malaysia (UKM), Kuala Lumpur, Malaysia; 2 Endocrine Hypertension, Department of Clinical Pharmacology and Precision Medicine, William Harvey Research Institute, Queen Mary University of London, London, United Kingdom; 3 St Bartholomew’s Hospital, Barts Health NHS Trust, London, United Kingdom; 4 NIHR Barts Biomedical Research Centre, Barts and The London School of Medicine and Dentistry, Queen Mary University of London, London, United Kingdom

## Abstract

Primary aldosteronism is the most common single cause of hypertension and is potentially curable when only one adrenal gland is the culprit. The importance of primary aldosteronism to public health derives from its high prevalence but huge under-diagnosis (estimated to be <1% of all affected individuals), despite the consequences of poor blood pressure control by conventional therapy and enhanced cardiovascular risk. This state of affairs is attributable to the fact that the tools used for diagnosis or treatment are still those that originated in the 1970–1990s. Conversely, molecular discoveries have transformed our understanding of adrenal physiology and pathology. Many molecules and processes associated with constant adrenocortical renewal and interzonal metamorphosis also feature in aldosterone-producing adenomas and aldosterone-producing micronodules. The adrenal gland has one of the most significant rates of non-silent somatic mutations, with frequent selection of those driving autonomous aldosterone production, and distinct clinical presentations and outcomes for most genotypes. The disappearance of aldosterone synthesis and cells from most of the adult human zona glomerulosa is the likely driver of the mutational success that causes aldosterone-producing adenomas, but insights into the pathways that lead to constitutive aldosterone production and cell survival may open up opportunities for novel therapies.

## Introduction

**Fig. 1:**
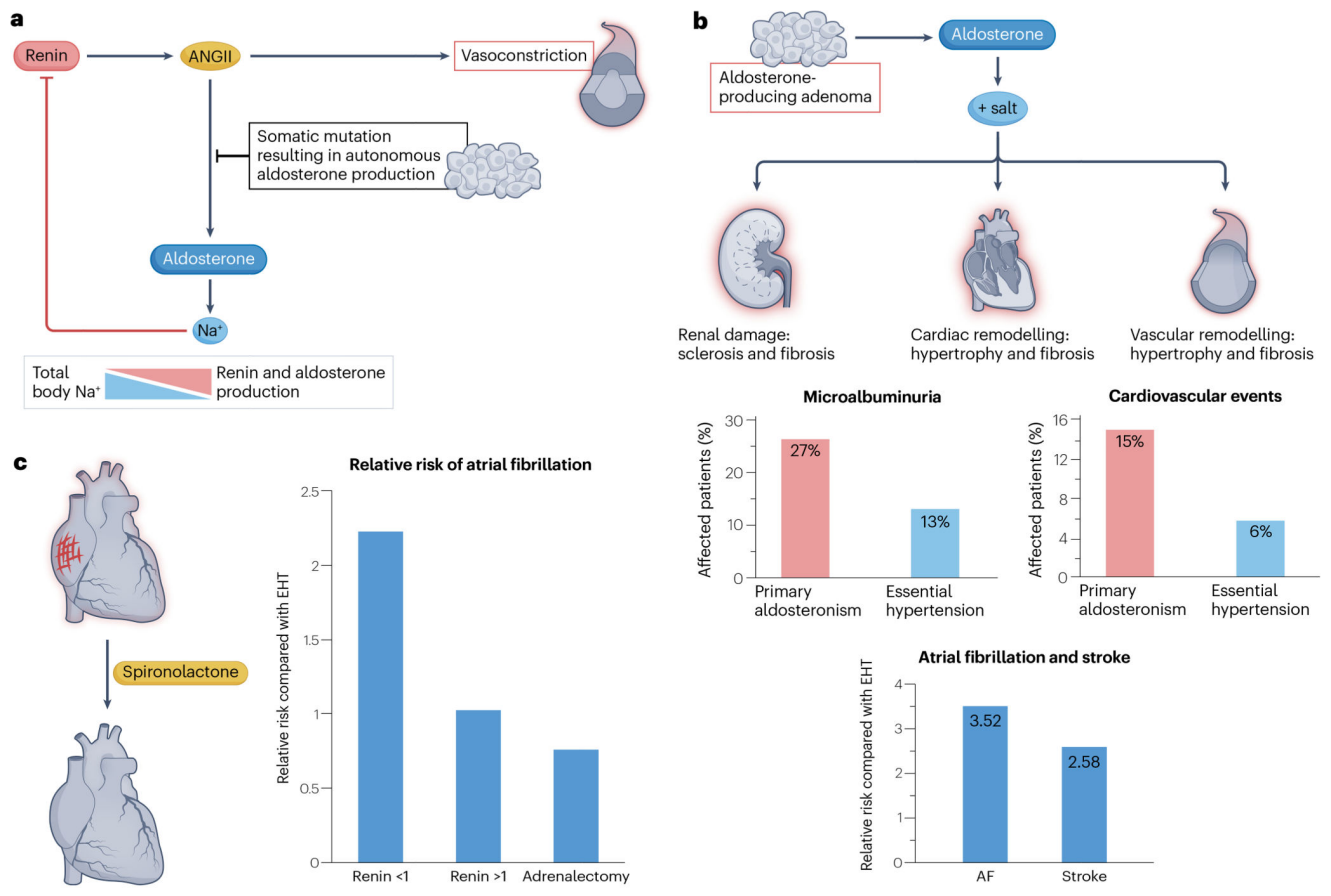
The cause, impact, and reversal with treatment, of sporadic primary aldosteronism. **a**, In sporadic primary aldosteronism, somatic mutations result in autonomous aldosterone production, which leads to excess Na^+^retention by the kidneys. These in turn detect a high Na^+^flux through the Na^+^-K^+^−2Cl^-^ transporter in juxtaglomerular macula densa cells, which signal to the adjacent renin-producing cells of the afferent arteriole to switch off renin secretion. The net consequence is a sustained elevation of plasma aldosterone and suppression of plasma renin and angiotensin II (ANGII). **b**, Autonomous aldosterone production in the presence of salt is associated with continuous renal Na^+^ reabsorption despite the suppression of ANGII and renin. Such inappropriate sodium retention leads to renal damage, cardiac remodelling, and vascular remodelling^10,12^. Patients with primary aldosteronism are at a higher risk than patients with essential hypertension of kidney and cardiovascular damage, detected as events such as atrial fibrillation and stroke. Relative risk data for atrial fibrillation and stroke are taken from Monticone et al.^12^. **c**, The effects of aldosterone can be reversed with the use of competitive mineralocorticoid receptor antagonists (MRAs), such as spironolactone, which inhibit the reabsorption of Na^+^ in the distal convoluted tubule and collecting duct. The inhibition of Na^+^ retention can be detected as a rise in plasma renin. In patients whose renin is adequately de-suppressed with use of MRAs, tissue damage by aldosterone and salt is prevented or reversed, attenuating the increased risks of cardiovascular and renal events^11,191^. Relative risk data for atrial fibrillation following MRA treatment or adrenalectomy are taken from Hundemer et al.^191^. AT1R, ANGII type 1 receptor. Graphs showing the percentage of patients affected by microalbuminuria and cardiovascular events in part **b** are adapted with permission from ref. 10, Elsevier.

Within a year of the discovery of aldosterone in 1953, a description of the first patient with primary aldosteronism (PA) signalled a dual trajectory for clinical and research interest in the hormone^1,2^. Over the next 70 years, many of its intertwined physiological and pathological roles have become clear (Fig. 1). For much of this period, the clinical syndrome of PA has held the status of an endocrine curiosity. Perhaps because of the dramatic clinical features of the index case described by Jerome Conn (sustained, severe hypertension, marked hypokalaemia and an adrenal tumour large enough to be seen by adrenal venography)^2^, PA was for many decades after Conn’s death considered a rare, relatively benign form of hypertension only to be suspected if the patient was profoundly hypokalaemic^3–7^. However, the characterization of PA as a rare, benign disease with a requirement for hypokalaemia is now acknowledged to be incorrect.

Over the past decade, clinical and molecular studies have catapulted PA onto the centre stage of cardiometabolic research. First, several rigorous clinical studies that used plasma aldosterone-to-renin ratio (ARR), rather than absolute thresholds of plasma aldosterone itself, indicated prevalence rates for PA of 5–11% in unselected hypertensive populations – a range that is uncannily similar to Conn’s prediction of 10%^8–10^. On top of these prevalence figures, the observation that the cardiometabolic consequences of PA (including stroke, atrial fibrillation, renal impairment and ischaemic heart disease) exceeded that of essential hypertension by a ratio of at least 2:1, provides an impetus to advance screening strategies and targeted therapies for PA^11,12^. We and others have estimated that fewer than 1% of cases of PA are detected and treated^13,14^; this underdiagnosis combined with the excess morbidity attributed to PA makes it difficult to exaggerate the public health consequences of this shortfall in clinical care.

Through molecular studies, it became clear that a substantial proportion of unilateral aldosterone-producing adenomas (APAs), which account for 30–50% of all cases of PA, harbour a gain-of-function somatic mutation^15–17^. The landmark report in 2011 of mutations in the K^+^-channel gene, *KCNJ5* (ref. 18), heralded a rich vein of molecular research. Recurrent somatic mutations in several additional genes were soon discovered, along with a larger number of upregulated genes in APAs or in the aldosterone-producing cells of the adrenal zona glomerulosa (ZG) from which APAs were presumed to arise. Understanding of the molecular signature of most APAs was enhanced by the development of selective and specific antisera for the steroidogenic enzymes^19^, and through the discovery of gene expression patterns that were unique to subsets of APAs^20–23^. These genotype–phenotype studies, combined with the finding that APA size correlates inversely with the expression of *CYP11B2* (which encodes aldosterone synthase)^24^, led to the realization that the ‘classic Conn’s tumour’ manifestation of PA is the exception rather than the rule. This realization was reinforced by the paradox that the cellular appearance of these ‘classic’ APAs resembles the cortisol-producing zona fasciculata (ZF) rather than ZG^17,18,21,25^.

**Fig. 2:**
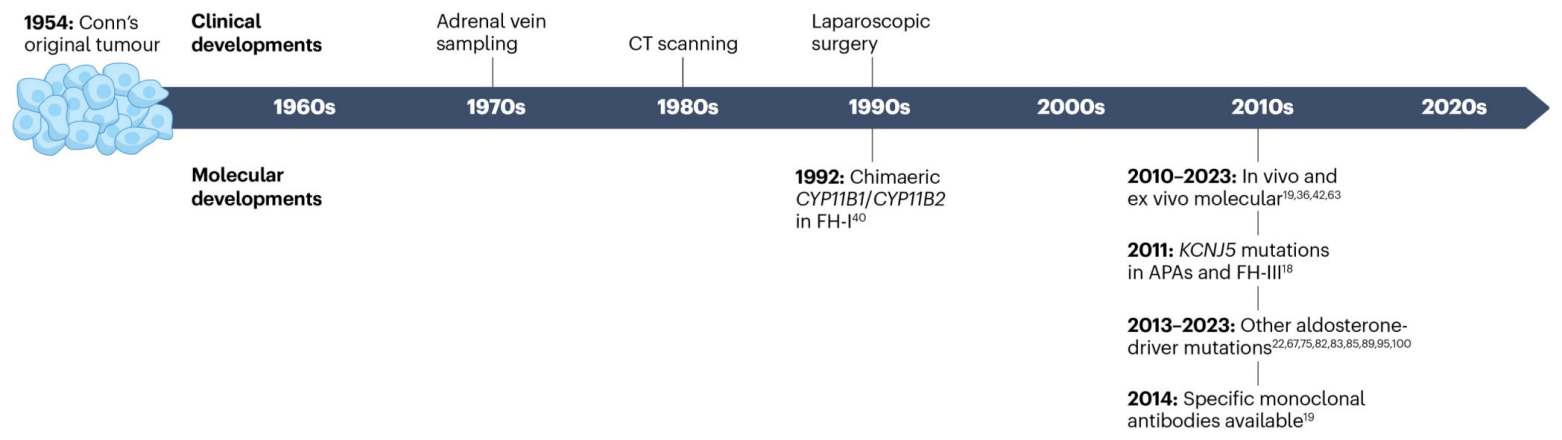
Timeline of clinical and molecular advances in primary aldosteronism. Seventy years after the first description of Conn’s syndrome, now known as primary aldosteronism, standard clinical practice for its management remains the removal of the entire adrenal, regardless of lesion size. Standard techniques include adrenal vein sampling, CT scanning and laparoscopic surgery. However, a number of key molecular scientific discoveries in the last 30 years have provided insights into the molecular pathogenesis of primary aldosteronism, and may identify new therapeutic approaches^18,19,22,36,40,42,63,67,75,81–83,85,89,95,100^. APA, aldosterone-producing adenoma; FH, familial hyperaldosteronism

These discoveries herald a new era in PA research. The clinical studies highlight the public health challenge posed by PA – that is, how to recognize the >99% of patients who currently elude diagnosis. The molecular studies suggest a potential means with which to meet this challenge. Clinically, little has changed in the 30, 40 and 50 years since the advent of laparoscopic surgery, CT scans and adrenal vein sampling (AVS), respectively^26–29^ (Fig. 2). The entire adrenal gland is still removed for the sake of a 1-cm benign lesion. The contrast of this approach with that of another such lesion – the peptic ulcer – could hardly be more striking, where over a similar period of 50 years, curative therapy progressed from gastrectomy to a week of drug therapy. If science can identify the ‘helicobacter’ of PA, we can surely look for its ‘omeprazole’ as a medical cure.

In this Review, we focus on molecular discoveries and developments that have the potential to transform the clinical management of patients with PA. We describe emerging innovations in diagnosis and therapy that may – in the future – be tailored to permit optimal management of each patient. We appreciate that many of the innovations and discoveries have been made in patients undergoing investigation or treatment for unilateral PA, who are a fraction of the total and are furthest in line from primary care, where the main bottleneck of diagnosing PA resides. However, interest in them is justified partly because they are the patients in whom links between molecular pathogenesis and outcomes are most readily studied, and partly because the current arduous course of diagnosing and managing their disease discourages physicians from seeking such patients^14^. We culminate in a hypothesis that draws on public health and molecular medicine to bridge the physiological and pathological links between Na^+^ and aldosterone by suggesting that PA arises – at least in part – as a maladaptive response to the chronic over-ingestion of salt.

## Defining primary aldosteronism

PA is the most common cause of endocrine arterial hypertension and is characterized by the autonomous production of aldosterone from one or both adrenal glands. The autonomy of this disorder contrasts with normal physiological processes, and with secondary aldosteronism, in which aldosterone secretion is regulated largely by angiotensin II (ANGII). As the production of ANGII is dependent on the secretion of the enzyme renin, the practical definition of PA refers to the ratio of aldosterone and renin measured in blood^30^. Like many other medical conditions defined by a threshold value within a quantitative continuum, the application of this definition is straightforward only for the minority of patients who reside near the extreme end of the continuum. When the continuum is a ratio, in this case the ARR, in which the culprit hormone (aldosterone) is the arithmetically distributed numerator, and the denominator a log-normally distributed sodium sensor (renin), the problems of diagnosis are multiplied: almost any measurable value of aldosterone in the presence of an undetectable plasma renin creates an ARR that is apparently diagnostic of PA. In practice, however, ARR frequently fails as a standalone measure of PA, and the autonomy of aldosterone secretion needs to be confirmed by showing that aldosterone is not suppressed (below another threshold value) by administration of salt or captopril^29^.

The pathogenesis of hypertension in PA can be ascribed to a combination of inappropriate sodium retention by epithelial cells of the kidneys (and consequent extracellular fluid volume expansion), together with the vasoconstricting actions of aldosterone^31^. The word ‘inappropriate’ is important; high levels of aldosterone are homeostatically logical in states of sodium depletion, but in the context of sodium excess can cause tissue injury, especially in the heart and kidney, as has been reviewed elsewhere^32^. In addition to ANGII, other determinants of aldosterone secretion under physiological conditions are extracellular potassium and adrenocorticotrophic hormone (ACTH)^33^.

The definition of PA as outlined by the Endocrine Society emphasizes the inappropriateness of aldosterone production for total body sodium status and for the prevailing levels of ANGII and blood potassium^29^. In practice it is not easy or routine to measure total body sodium status or ANGII concentrations, and blood potassium concentrations are not always a reliable indicator of total body potassium levels. Indeed, the measurement of aldosterone itself is also not without controversy, and much higher prevalence figures for PA might be obtained if 24-h urine measurements were routinely used to avoid diurnal fluctuations in secretion^34^. However, the definition is pertinent to our hypothesis that the pathogenesis and frequency of PA relates – at least in part – to a maladaptive response to high sodium intake.

Prior to the availability of molecular and genetic insights, PA was believed to result from either a single, unilateral, benign APA or a bilateral idiopathic adrenal hyperplasia (IAH). Clinical subtyping investigations, cross-sectional imaging and AVS, were used in an attempt to distinguish these two pathological conditions, with a view to applying laparoscopic surgery for the treatment of APA and medical management for IAH (as reviewed elsewhere^35^). However, in reality, no clinical pathological criteria define either APA or IAH. The standard, historical approach to the diagnosis of APA was to perform classic haematoxylin and eosin (H&E) staining of the resected adrenal gland to detect the presence of one or more nodules (thereby raising suspicion of an APA) and then to identify the predominant cell type. Some nodules were reported to have small, compact cells; some to have large, foamy, lipid-rich cells; and some to have a mixture of the two. The development of highly specific antibodies to the enzymes responsible for the final step of cortisol and aldosterone synthesis (11-β hydroxylase and aldosterone synthase, encoded by *CYP11B1* and *CYP11B2*, respectively) facilitated a link between descriptive pathology and functional activity, and uncovered a spectrum of unilateral PA manifestation that included unilateral micro- or macronodular hyperplasia^19,36,37^. In patients with bilateral PA, in whom conventional pathology reported gross hyperplasia, immunohistochemistry showed mainly focal rather than diffuse expression of aldosterone synthase^38^. However, a decade on, only a few hospitals perform routine CYP11B2 immunohistochemistry of adrenal glands removed from patients with PA.

## Germline mutations

PA can be categorized on the basis of its heritability. The vast majority of cases of PA are sporadic but, rarely, the condition affects multiple members of the same family and is termed familial hyperaldosteronism (FH). Various forms of FH exist, distinguished by their clinical features and reported genetic causes. The characteristic early onset and heritability of FH makes it an attractive target for understanding the more common sporadic PA; conversely, large-scale next-generation sequencing studies of sporadic cases has greatly informed the FH field. All of the Mendelian causes of PA are rare, and specialists within different regions often find that different members of a handful of families are referred to them. Although familial cases represent a tiny proportion of PA, investigations of these patients have shown that PA can be caused by mutations that induce constitutive aldosterone production (Table 1).

## Somatic mutations

In contrast to germline mutations, which are rare and unlikely to be seen by most hypertension specialists, somatic mutations that cause PA are among the most common recurrent mutations seen in medicine. Indeed, a systematic analysis of non-silent somatic mutation rates across organs found a significant excess in the adrenal gland, similar to that of skin^39^. Most of these mutations are likely to be non-functional – for example, representing products of DNA damage by reactive oxygen species generated during incomplete mitochondrial steroid oxidations. However, in some instances, gain-of-function mutations that drive constitutive aldosterone production might be beneficial to the mutated cells, and thus increase their frequency through the evolutionary selection of such mutations. In the following sections we describe some of the key findings of recurrent somatic mutations that have been linked to PA (Box 1).

## KCNJ5

**Fig. 3:**
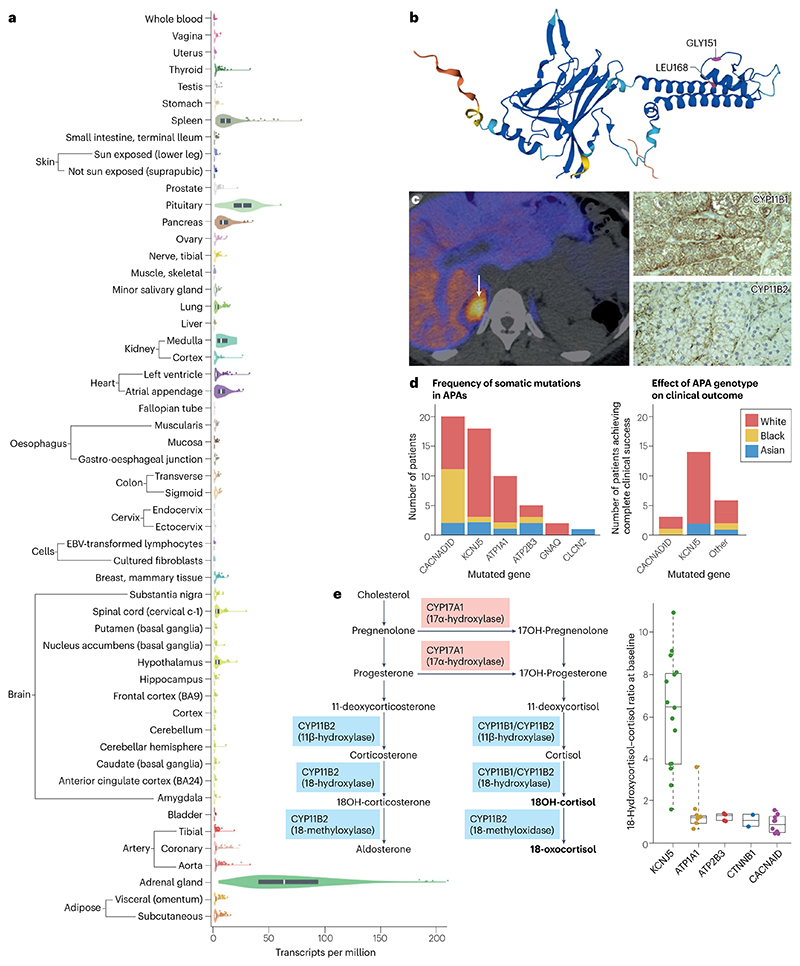
The contribution of *KCNJ5* mutations to the pathogenesis of primary aldosteronism. **a** Search for *KCNJ5* (ENSG00000120457.11) in the GTEx portal shows that the adrenal gland expresses the highest levels of this gene. Data are shown as transcripts per million, calculated from a gene model with isoforms collapsed to a single gene. No other normalization steps have been applied. Box plots are shown as median and 25th and 75th percentiles; points are displayed as outliers if they are above or below 1.5 times the interquartile range^140^. **b**, Structural analysis of the potassium channel Kir 3.4, which is encoded by *KCNJ5*, shows the originally described mutations (Gly151Arg and Leu168Arg) within or close to the selectivity pore of the channel^18^. **c**, A typical 11C-metomidate PET-CT scan in a patient with a *KCNJ5*-mutant APA (arrow). Immunohistochemistry of tissue sections from mutant APAs showed them to more closely resemble cortisol-producing (zona fasciculata; ZF) than aldosterone-producing (zona glomerulosa; ZG) adrenal cells, with more striking staining for CYP11B1 than CYP11B2. **d**, Data from the MATCH study indicate that *KCNJ5* mutations are the most common mutations found in APAs from self-identified white individuals^63^. Data from MATCH also indicate that APA genotype is indicative of complete clinical success following adrenalectomy, with removal of *KCNJ5*-mutated APAs most likely to result in complete clinical success. The colours represent self-reported ethnicity. **e**, The presence of ZF- and ZG-specific enzymes (CYP17A1 and CYP11B2, respectively) in the same cell of a *KCNJ5*-mutant APA indicates that such cells can synthesize the ‘hybrid’ steroids 18-oxocortisol and 18-hydroxy-cortisol (18-OH-cortisol) that a normal ZG or ZF cell alone would not be able to produce. Data from the MATCH study have demonstrated the potential diagnostic utility of hybrid steroids as a metabolic marker of a *KCNJ5*-mutated APA^63^. Part **a** reproduced from ref. 192, CC BY 4.0 (https://creativecommons.org/licenses/by/4.0/). Part **b** reproduced from ref. 193, CC BY 4.0 (https://creativecommons.org/licenses/by/4.0/). Parts **d** and **e** adapted from ref. 63, CC BY 4.0 (https://creativecommons.org/licenses/by/4.0/).

The field of molecular genetics in PA started with Lifton’s discovery of the *CYP11B1/CYP11B2* chimaeric gene in families with GRA^40^ (Table 1); however, this finding did not impact our understanding of sporadic PA until his landmark identification, 19 years later, of somatic mutations in *KCNJ5* (ref. 18). These mutations were found by whole exome sequencing of four relatively large APAs, each containing a Gly151Arg or Leu168Arg mutation. These findings were replicated and, in all, 8 out of 22 APAs were found to have one of the mutations^18^. *KCNJ5* encodes the Kir3.4 potassium channel and the two mutations both affect residues at or near its K^+^-selectivity channel (Fig. 3). An important link between Lifton’s 1992 and 2011 discoveries are his report, published shortly before *KCNJ5* identification, of a family with Mendelian ‘non-glucocorticoid remediable aldosteronism’^41^. A germline mutation (Thr158Ala) in *KCNJ5* was subsequently found in the father and two daughters, whose huge adrenals with bilateral hyperplasia required surgical removal to control severe hypertension. A feature of this family was that not only aldosterone but also cortisol was unsuppressed by dexamethasone, despite the absence of clinical features of hypercortisolism. In common with GRA families, the sisters demonstrated high excretion of the hybrid steroids, 18-oxocortisol and 18-hydroxycortisol. Subsequent investigations of patients with sporadic PA have similarly revealed that a subset demonstrates incomplete suppression of serum cortisol in response to dexamethasone pre-adrenalectomy, and that the APAs in this subset of patients have a *KCNJ5* mutation^21,41–44^.

The Kir 3.4 potassium channel is highly expressed in aldosterone-producing cells of the ZG and contributes to hyperpolarization of the ZG cell membrane^45^. Indeed, had transcriptome data been as widely available in 2011 as it is now, *KCNJ5* might have been an obvious candidate gene to test for mutations, given its strikingly high and selective expression in the human adrenal gland (Fig. 3). Its absence from the adrenal glands of mice, in which models of PA had centred on the potassium leak channels KCNK3 and KCNK9, are an illustration of why human datasets have largely proven more valuable than rodent studies for clues to the pathogenesis of hypertension^46^. The occasional patients with germline mutations or mosaicism of *KCNJ5* highlight the potential impact of non-adrenal mutant *KCNJ5* expression, particularly in the heart and pituitary gland, where even low-level expression could contribute to arrhythmias and reduced dexamethasone suppression of cortisol, respectively^41,47^. Cells transfected in vitro with the Gly151Arg or Leu168Arg Kir 3.4 mutants demonstrated loss of selectivity for potassium, increased permeability to sodium and ZG membrane depolarization^18^. The resultant influx of calcium activates aldosterone synthesis and secretion. Further *KCNJ5* mutations have subsequently been documented within or near to the potassium selectivity filter (between Glu145 and Leu168), with very few exceptions, but the originally described mutations account for the vast majority of mutant *KCNJ5* (refs. 18,48–53).

A set of somatic *KCNJ5* mutations, which also occasionally appear in the germline – for example, Thr158Ala, Gly151Arg and Glu145Gln – cause a florid Mendelian syndrome with macroscopic adrenal hyperplasia, early-onset hypertension and hypokalaemia^18,54,55^. By contrast, mutations that are unique to the germline (for example, Gly151Glu and Tyr152Cys) cause a milder clinical phenotype that responds readily to treatment with mineralocorticoid antagonists^54,56,57^. Functional studies suggest that these germline-only variants cause more profound sodium permeability than other variants, which may result in cell death and hence prevent the development of hyperplasia^54^.

### Genotype-phenotype subtyping of KCNJ5 mutant APAs

As the first gene found in sporadic PA, *KCNJ5* mutant APAs became the most extensively characterized genotype^15,58^. The estimated prevalence of *KCNJ5* mutations in resected APAs is 43%; however, the reported range is broad (12–80%), with higher frequencies reported in Japan and China than in European countries, and a lower frequency in one study of African Americans^58–60^. Whether these reported variations reflect true differences in prevalence or selection bias remains unclear. Somatic *KCNJ5* mutations in APAs are more frequent in women than in men, except possibly in Asian cohorts, and present at a younger age than other APA genotypes^58,59,61,62^ (Fig. 3). In our own analysis, we identified *KCNJ5* mutations in 43% of APAs, and we found a strong genotype–phenotype pattern, not only in age and gender but also in several aspects of pathology and gene expression^21^. On H&E-stained sections, the cells resembled those of adrenal ZF rather than ZG, and on both quantitative (q)PCR and semi-quantitative immunohistochemistry, we observed higher expression of *CYP11B1* than *CYP11B2*, and high levels of *CYP17A1*, which encodes the obligatory enzyme in cortisol synthesis, 17-α-hydroxylase. The overall picture led us to describe the *KCNJ5*-mutant APAs as ZF-like, and most of the non*-KCNJ5*-mutant APAs as ZG-like^21^. In retrospect, it seems remarkable that the ‘classic Conn’s tumour’, which is typically 1.5–2.5 cm in diameter, is composed of cells that resemble the lipid-rich, cortisol-secreting cells of ZF, rather than the compact cells of ZG. We now realize that such a ZF phenotype is uncommon except in *KCNJ5* mutant APAs^21,63^. *KCNJ5*-wild type APAs most commonly have compact cells which, except for their rounded nuclei, resemble ZG cells. The ZG-type of APA is often small (0.5–1.5 cm in diameter, and hence commonly missed on CT), but stains more densely for aldosterone synthase than the *KCNJ5*-mutant, ZF-like APAs. *KCNJ5*-mutant APAs also have a lower density of KCNJ5 itself than ZG-like APAs; this finding may sound paradoxical, but it is consistent with the lower expression of KCNJ5 in the ZF than the ZG and the overall ZF-like phenotype of *KCNJ5*-mutant APAs.

We initially hypothesized that the phenotype of *KCNJ5*-mutant APAs pointed to an origin in the outer ZF rather than the ZG, and that such mutations in a ZG cell with abundant KCNJ5 would mimic the outcome of HEK293T cells transfected with Kir 3.4 containing the electrophysiologically strongest (Gly151Glu) mutation; that is, rapid cell death by Ca^2+^ overload^54^. However, extensive transcriptome analyses have revealed more similarities than differences between ZF-type and ZG-type APAs^22,64^. The likely inference from these findings is that the ZF-like cells in *KCNJ5*-mutant APAs reflect the consequence of, rather than origin of the mutation, and that the mutation of *KCNJ5* in a ZG cell recapitulates the changes that occur spontaneously during ZG cell transition or migration into the ZF^64,65^. Primary ZG cells are difficult to transfect, but in one experiment we found that transfection of mutant *KCNJ5* switched off the gene *NEFM*, which encodes neurofilament M – a key marker of ZG cells, supporting the notion that the ZF-like phenotype of *KCNJ5* mutant APAs is a consequence of its mutation, as opposed to the mutation originally arising in ZF cells^66^.

## ATP1A1 and ATP2B3

**Fig. 4:**
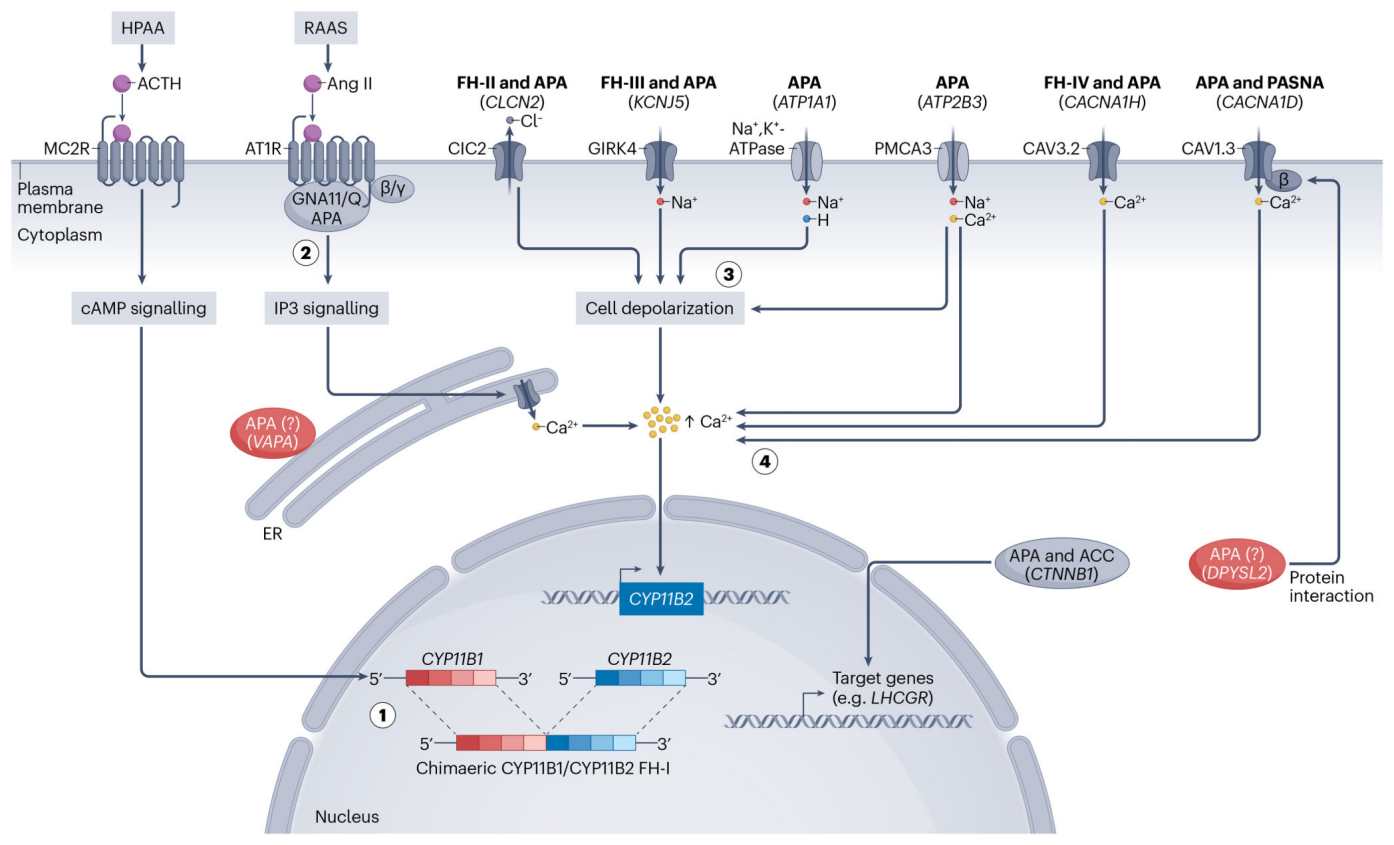
Germline and somatic mutations associated with primary aldosteronism. Pathological autonomous aldosterone production in a cell can occur by a number of different mechanisms. (1) In familial hyperaldosteronism (FH)-I, the *CYP11B1/CYP11B2* hybrid gene is activated by cAMP signalling, which occurs when adrenocorticotropic hormone (ACTH) binds to the melanocortin 2 receptor (MC2R) in response to hypothalamic–pituitary–adrenal axis (HPAA) activation. (2) *GNA11* and *GNAQ* mutations in aldosterone-producing adenomas (APAs) prevent termination of G protein signalling downstream of the renin–angiotensin–aldosterone system, which is activated when angiotensin II (ANGII) binds to the angiotensin 1 receptor (AT1R)), leading to increased calcium release from intracellular stores via the inositol trisphosphate (IP_3_) signalling pathway. These mutations co-occur with *CTNNB1* mutations, which are found in APAs and adrenocarcinomas, and prevent β-catenin degradation, leading to increased transcription of target genes such as *LHCGR*. (3) *CLCN2* mutations in FH-II and APAs, *KCNJ5* mutations in FH-III and APAs, and *ATP1A1* and *ATP2B3* mutations in APAs lead to abnormal permeabilities for Cl^-^ Na^+^, H^+^, and Ca^2+^. These abnormalities cause cell depolarization, which increases intracellular calcium concentrations, thereby stimulating *CYP11B2* expression and aldosterone production. Acidification and an impaired pump function may also have a pathological role (not shown). (4) *CACNA1H* mutations in FH-IV and APAs and *CACNA1D* in primary aldosteronism with seizures and neurological abnormalities (PASNA) and APAs increase calcium permeability by stimulating the calcium signalling pathway directly. High-probability pathogenic somatic mutations have also been found in *DPYSL2* and *VAPA. DPYSL2* is highly abundant in the zona glomerulosa and has previously been linked to ion-channel trafficking. Based on sequence prediction, we hypothesize that it may bind to the β-subunit binding site of Cav1.3 (refs. 63,171). *VAPA* is also linked to protein trafficking and is thought to be expressed on the endoplasmic reticulum where IP3 signalling acts on the calcium channel to release calcium from intracellular stores.

The identification of a *KCNJ5* genotype–phenotype correlation prompted a search for further mutations in archived APA tissue. Comparisons of whole exome sequencing data from APAs without the *KCNJ5* mutation and/or with ZG morphology, with adjacent normal adrenal tissue, led to the discovery of somatic mutations in *ATP1A1* and *ATP2B3* (encoding the α-1 subunits of Na^+^/K^+^-ATPase and Ca^2+^-ATPase3, respectively)^22,67^ (Fig. 4). Mutations in these two genes account for 5–20% of all APAs and are more common in white people than in other populations^16,17^.

The *ATP1A1*-encoded Na^+^/K^+^-ATPase is a member of the P-type ATPase family. The catalysed exchange of three cytoplasmic sodium ions for two potassium ions by this ATPase generates electrochemical gradients that maintain resting membrane potential and cellular excitability. The α-subunit consists of ten transmembrane domains (M1–M10) with intracellular N- and C-tails. Various point mutations and deletions in *ATPA1A* have been described, most involving disruption of a leucine at position 104, or a glutamic acid at position 334 – regions that juxtapose to form the key area for binding of sodium and potassium. It was initially thought that alterations in the affinity of the pump for sodium and potassium resulted in a loss of function leading to abnormal depolarization, opening of voltage-gated calcium channels and augmented aldosterone synthesis following increases in intracellular calcium concentrations^67^. However, *ATP1A1* mutations have now been demonstrated to increase aldosterone production through a variety of mechanisms including a gain of function^22^. This suggests that some genes need to achieve both gain and loss of function in order to overcome pathways that inhibit aldosterone production^68,69^. To date, no germline mutations in *ATP1A1* have been reported to cause PA, although rare cases have been described of germline *ATP1A1* mutations causing magnesium wasting and abnormalities of the central nervous system (for example, spastic paraplegia or the Charcot–Marie–Tooth-like disorder)^70,71^.

The *ATP2B3*-encoded plasma membrane calcium ATPase (also known as PMCA3) belongs to the same family as the Na^+^/K^+^-ATPase and is similarly composed of ten transmembrane segments. PMCA3 removes one cytosolic Ca^2+^ in exchange for two H^+^ and has a key role in the maintenance of intracellular calcium homeostasis. Several mutations in *ATP2B3* have been described: all involve deletions of a least 2 amino acid residues in an area that is essential for calcium ion binding within the M4 domain^16,17,60,67,72^. The deletions result in membrane depolarization; abnormally increased permeability to cations, notably sodium and calcium; opening of voltage-gated calcium channels; and augmented CYP11B2 activity^73^. Germline mutations in *ATP2B3* cause X-linked congenital cerebellar ataxia, but not in association with PA^74^. The widespread, fundamental role of ATPases makes them an unattractive target for pharmacological modification in the context of PA.

## CACNA1D

**Fig. 5:**
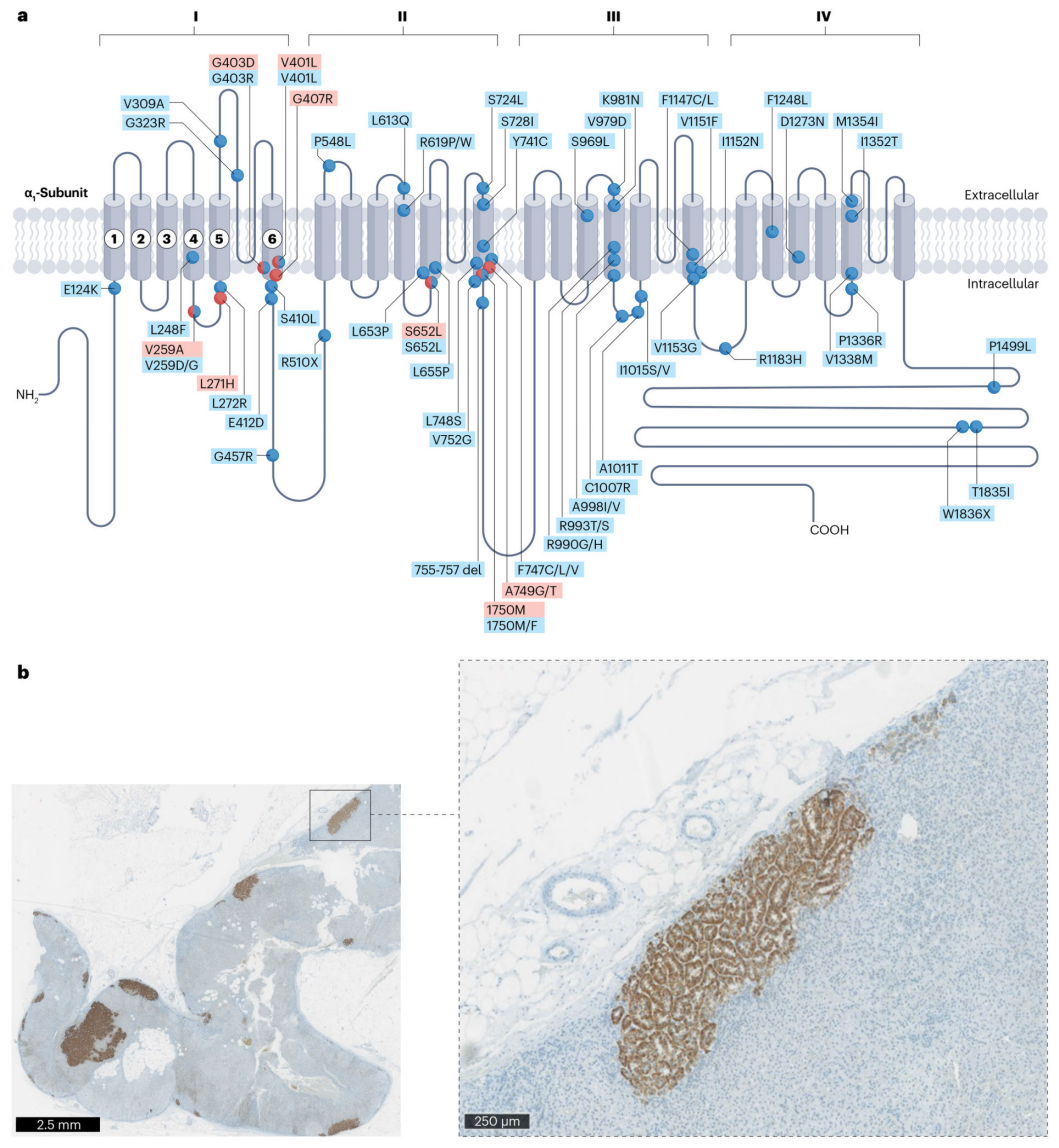
The plethora of *CACNA1D* mutations associated with aldosterone-producing adenomas and aldosterone-producing micronodules. **a**, *CACNA1D*, which encodes the pore-forming α1 subunit of a voltage-gated calcium channel (Cav1.3), is the most commonly mutated gene in aldosterone-producing adenomas (APAs) and aldosterone-producing micronodules (APMs) from patients with primary aldosteronism who self-identify as Black or who have bilateral adrenal hyperplasia. The Ca_v_1.3 α1-subunit comprises four homologous repeats (I–IV), each comprising six transmembrane segments (S1–S6). Germline mutations (red circles) and somatic mutations (blue circles) have been identified throughout regions I–IV. More than 70 mutations have been described, mostly in small APAs or APMs^194^. The majority of mutations in *CACNA1D* with evidence of pathological function are in conserved sites within functional domains, such as the voltage-sensing domain, the channel-activation gate and the cytoplasmic S4-S5 linker, which couples the voltage-sensing domain to the pore. **b**, The identification of APMs (and small zona glomerulosa (ZG)-like APAs) was facilitated by the development of a specific monoclonal antibody for CYP11B2, which discriminated it from the highly abundant and homologous CYP11B1 (ref. 19). These APMs and small ZG-like APAs commonly harbour a *CACNA1D* somatic mutation^22,107^.

Whole-exome sequencing of ZG-like and/or *KCNJ5* wild type APAs also led to the identification of gain-of-function somatic mutations in *CACNA1D*, which encodes the voltage-gated L-type Ca^2+^ channel, Ca_v_1.3 (refs. 22,75). This channel consists of repeat domains, numbered I–IV, each of which has six transmembrane segments (S1–S6) and a membrane-associated loop between the S5 and S6 segments^76^. Of importance to channel activation are segments S4–S6, as S4 is involved in voltage sensing, whereas S5, S6 and the membrane-associated loop form the pore lining^77^. More than 70 mutations have been described in *CACNA1D* so far, and their overall reported prevalence in APAs in most reports has been around 9%^38,78^ (Fig. 5). However, our own experience, using PET CT to visualize smaller APAs, showed 9% to be a marked under-estimate, and below we discuss how *CACNA1D* mutations pre-dominate among somatic mutations found in patients with PA and non-adenomatous ZG.

The majority of mutations in *CACNA1D* occur in segments S4–S6, where they affect the function of the voltage-sensing domain, the channel activation gate and coupling of the voltage-sensing domain to the pore^75^. Patch clamping of cells transfected with mutant Ca_v_1.3 channels show that calcium influx is increased by various mechanisms, including a shift in voltage-dependent activation towards more negative voltages, a deceleration in channel inactivation and/or an increase in current through a higher open channel probability. Two of the originally identified mutations (that is, p.Gly403Arg and p.Ile770Met) were simultaneously reported in paediatric patients presenting with an extreme phenotype of PA, seizures and neurological abnormalities^75^. In both of these patients, antagonism of the calcium channels resulted in remission of hypertension and PA^79^.

### Rare recurrent aldosterone-driver mutations

#### CACNA1H and CLCN2

A small subset of unilateral APAs have been reported to harbour recurrent somatic mutations in *CACNA1H* (Ile1430Thr, Val1937Met)^80,81^, in the same region as the germline mutations that cause FH type IV (*CACNA1H* Met1549Val and Met1549Ile^82^; Table 1). *CACNA1H* encodes the pore-forming α1H subunit of the voltage-dependent T-type calcium channel Ca_v_3.2, which, similar to *CACNA1D*, is expressed in the adrenal ZG^82^. Also like *CACNA1D*, mutations in *CACNA1H* induce calcium influx, which increases *CYP11B2* mRNA levels and thus enhances aldosterone secretion. Yet, the prevalence of somatic *CACNA1H* mutations in APAs seems to be low, more similar to that of *ATP2B3* mutations^81^. However, most studies have only sequenced selected exons or hotspot regions of *CACNA1H* owing to its large size, and thus may have not thoroughly investigated the prevalence of pathogenic somatic mutations in this gene.

A similarly rare cause of sporadic PA is somatic mutations in *CLCN2*. These mutations occur in the same regions in which a germline mutation (Gly24Asp or Asn356_Leu359del) is associated with FH type II^83–86^ (Table 1). *CLCN2* encodes the inwardly rectifying chloride channel, CIC-2. The expression of mutant CLCN2 leads to depolarization of the adrenal cell membrane and activates the voltage-gated calcium channel, leading to Ca^2+^ influx, which in turn activates *CYP11B2* transcription^83,85^.

#### CTNNB1, GNA11/Q and CADM1

Although most somatic mutations in APAs are in genes that encode ion channels or transporters, whole-exome sequencing of 10 ZG-like APAs identified one APA with an exon 3 mutation in *CTNNB1* (ref. 22), which encodes β-catenin – a protein that is important for a number of processes, including cell differentiation. The index patient harbouring this mutant APA, and her first replicate both presented with PA in early pregnancy^22,87^. The putative relevance became apparent from scrutiny of their APA transcriptomes, which revealed an >100-fold increased expression of the gene that encodes the receptor for the pregnancy hormone, *LHCGR*, compared with levels in other APAs^87^. However, pregnancy and high LHCGR expression have not been consistently seen in subsequent reports^88^.

Whole-exome sequencing of an APA from a boy who presented with PA at puberty identified a somatic mutation in *CTNNB1* that co-existed with a mutation at Gln209 in *GNA11*. This combination was coincidentally also found in 2 of 40 consecutive APAs that we analysed at the same time^89^, and in retrospect, was also reported in our index *CTNNB1*-mutant patient^22^. Further genotyping has identified a total of 16 patients with a double mutation of a phosphorylation site in exon-3 of *CTNNB1* (which results in β-catenin entering the nucleus and activating transcription) and the Gln209 residue of *GNA11* or the closely homologous *GNAQ* – the G-proteins that mediate aldosterone production in response to ANGII stimulation^89^. 15 of these 16 patients are women, most of whom presented in the first trimester of pregnancy; all patients achieved 100% complete clinical success following adrenalectomy. Exon 3 mutations in *CTNNB1* are frequent in malignant tumours, including adrenocarcinomas. Somatic p.Gln209 mutations in *GNAQ* cause most uveal melanomas, and, as mosaic mutations of *GNA11* or *GNAQ*, cause Sturge–Weber and other cutaneous syndromes^90–92^. Elevations in the expression of *LHCGR* were observed in 15 of the 16 women with double-mutant APAs, but not in any of the 8 women APAs with solitary mutations of *CTNNB1*. LHCGR is coupled to adenylate cyclase, and mediates a rapid onset of hyperaldosteronism when stimulated in aldosterone-producing cells^87,93^. The p.Gln209 residue of *GNA11* and *GNAQ* is analogous to the p.Gln227 residue of *GNAS*, which is mosaically mutated in McCune–Albright syndrome, defined by the presence of different genotypic variants among cells arising from a single zygote^94^. Some of the adrenal tissue adjacent to the double-mutant APAs was similarly mosaic. Our hypothesis for the strikingly high complete success rate of adrenalectomy in these patients is that these mutations arise embryogenically after the right or left adrenal primordium develops, such that there is no contralateral abnormality.

Whole-exome sequencing of the 40 consecutive APAs also identified another gene, *CADM1*, with a deleterious p.Val380Asp mutation within its single intramembranous domain^95^. *CADM1* (previously known as *SynCAM)* encodes a cell adhesion molecule, which is most highly expressed in the central nervous system, especially the cerebellum, where it has a role in synaptogenesis^96^. Five further patients, with either p.Gly379Asp or p.Val380Asp somatic mutations, were subsequently identified by whole exome or targeted sequencing of ~550 APAs^95^. Transfection of the mutations into adrenocortical cells led to upregulation of pathways linked to biological rhythm, with upregulation and downregulation, respectively, of the out-of-phase E-box and RORE clock genes. One of the RORE clock genes, *NPAS2*, which was upregulated in the index *CADM1*-mutant tumour, is a paralog of *CLOCK*. Like *CLOCK*, it dimerizes with genes involved in circadian rhythm, such as *BMAL1*, to drive the transcription of other clock genes (such as *CRY1–2* and *PER1–3*)^97,98^. Of interest, the two index cases (P1 and P2) with p.Gly379Asp or p.Val380Asp mutations in *CADM1* had apparently periodic secretion of aldosterone. This finding prompts the question of whether *CADM1* mutations may be more frequent (than our estimated 1%) among patients who elude diagnosis by conventional, random blood sampling. An unrelated clinical study coincidentally reported that measurement of 24-h urinary aldosterone secretion rather than a ‘snapshot’ measurement of ARR in blood, may reveal the prevalence of PA to be twice as high as the 5–11% estimate^34^. Serial blood measurements in PA, and microdialysis measurements of interstitial aldosterone, also point to great variability in aldosterone secretion, possibly following diurnal or ultradian rhythms^99^. The two patients with *CADM1*-mutant APAs who had documented periodicity of aldosterone secretion were the two who achieved complete clinical success. The explanation for this observation might again be related to true lateralization, with rare mutations being unlikely to arise bilaterally. An alternative explanation is that cumulative exposure of target tissue to aldosterone is lower in patients with variable than in those with sustained aldosterone excess.

#### Somatic deletion in a zinc transporter SLC30A1.

Discovery of recurrent somatic mutations in *SLC30A1* (ref. 100), which encodes the zinc transporter ZnT1, extends the number of ion channels or transporters linked to PA. The 51_57del variant caused abnormal Na^+^ influx, depolarization of the resting membrane potential and opening of voltage-gated calcium channels. As with other somatic mutations in APAs, the resultant increase in cytosolic Ca^2+^ signalling stimulated *CYP11B2* expression and aldosterone production.

### Insights from transcriptome analyses

The rank of the genes upregulated in APAs, compared with the adjacent adrenal, varies among studies, but marked upregulation of *CYP11B2, VSNL1* (encoding a putative transmembrane calcium-sensor), and *PCP4* (a Purkinje-cell gene), are consistent findings^20,101^,^102^. All three of these genes are also expressed, diffusely or in clusters, at higher levels in the ZG than in the ZF^102^,^103^.

Our own microarray of seven APAs that we considered to be ZF-like (all with *KCNJ5* mutations), seven ZG-like APAs and regions (ZG and ZF) adjacent to all 14 APAs (isolated using laser capture microdissection), identified striking differences between the transcriptomes of the ZF-like and ZG-like APAs^64^,^65^,^104–106^, which are corroborated by a similar laser capture microdissection-based microarray^107^ and by later RNA sequencing-based comparisons of APA transcriptomes^63,88^. Notably, levels of *CYP11B1* and *CYP17A1* were higher in the ZF-like APAs than in the ZG-like APAs. Second, a much larger number of genes were several-fold up-regulated in ZG-like APAs than the converse. Genes that were more upregulated in ZG-like APAs (that is the *ATP1A1* and *CACNA1D* mutants) compared with ZF-like APAs (the *KCNJ5* mutants) included *NPNT* (encoding nephronectin), *MYOM1* (encoding the heart-muscle protein, myomesin) and *KIAA1549L* (also known as C11orf41, encoding a neuronal gene of unknown function)^22,64^. Although *NPNT* is selectively expressed in the ZG, where its encoded protein binds to an integrin (α8, β1) around the glomeruli^106^, *MYOM1* and *KIAA1549L* are barely expressed in normal adrenal tissue. However, differences between ZF-like and ZG-like APAs were greatly out-numbered by similarities in their transcriptomes.

We have identified 40 genes with expression at least 4-fold higher in the ZG than in the ZF; indeed, 10–25-fold higher in the case of the top six genes (Supplementary Table 1)^104^. With the exception of *VSNL1*, none of the other ‘top genes’ in ZG were previously associated with aldosterone production^65,104,105,108^. Several have functional pedigrees in other tissues. For instance, *LGR5* and its ligand *RSPO3* provide Wnt-directed signals that control the migration of gut stem cells from the crypt to the intestinal surface^109^. In mice, *Rspo3* is required for adrenal development and adrenocortical cell replenishment from the capsule^110^, suggesting that *LGR5* could potentially have a role in controlling the centripetal migration of the adrenal cortex. However, the absence of *CYP11B2* from this list (Supplementary Table 1) corresponds to an almost complete absence of the protein from the ZG of adult adrenal tissue^19,111,112^. Interestingly, ex vivo expression of *CYP11B2* is inhibited by *LGR5* transfection, and its expression in vivo is inversely related to that of *LGR5* (ref. 105). Systematic pathway analysis of genes differentially regulated in ZG compared with ZF also indicated negative regulation of steroid and lipid synthesis, and of Wnt signalling^64,113^. These findings suggest that suppression of aldosterone secretion in adult ZG is, in part, an active process rather than a consequence of ANGII withdrawal due to excess of salt in the modern diet.

Differences in gene expression between ZF-like and ZG-like APAs were much smaller than differences between ZF and ZG^22,63,64^. Above, we inferred that ‘ZF-like’ refers to the pathology and biochemistry, but not the origins of the *KCNJ5*-mutant APAs. This inference is supported by the finding that several genes, such as *VAT1L* and *VCAN*, which are selectively expressed in the ZG (Table 1), were more highly expressed in the ZF-like than in the ZG-like APAs^104^. The converse set of genes (that is, those that were more highly expressed in ZG-like than in ZF-like APAs) were poorly expressed in adjacent adrenal tissue, and/or showed small differences in gene expression between normal ZF and ZG. This apparent anomaly was subsequently explained by the discovery of aldosterone-producing cell clusters (now known as aldosterone-producing micronodules, APMs).

### Aldosterone-producing micronodules

The vanishing *CYP11B2* paradox – that is, the disappearance of the enzyme required to synthesize aldosterone from the gland that makes the hormone – was largely resolved by the tour-de-force discovery of specific epitopes within the two highly homologous enzymes, 11-β hydroxylase and aldosterone synthase, which generated highly specific monoclonal antibodies for immunohistochemistry^19^. Numerous subsequent studies demonstrated that the diffuse expression of aldosterone synthase observed throughout the ZG of the paediatric adrenal gland was usually absent in adult ZG^114^. Instead, CYP11B2-positive cells were limited to discrete ~1–3-mm areas, which are now known as APMs, located beneath the adrenal capsule^36,115,116^ (Fig. 5b). Like the cells of most APAs they have rounded nuclei, unlike the dentate nuclei of physiological ZG cells.

Whether APMs should be considered a common precursor of ZG-like APAs, or a coincidental, physiological or pathological response to chronic salt excess is unknown. Excess salt, common in our modern diet, switches off the physiological production of aldosterone by suppressing the renin-angiotensin-aldosterone system, leading to decreased expression of aldosterone synthase (Fig. 1). If salt is responsible for the suppression of aldosterone synthase outside of APMs, there may be evolutionary advantage in the existence of a non-suppressible reserve of aldosterone synthase that enables constitutive aldosterone production, for example, to enable the production of transient ACTH-mediated increases in aldosterone in response to stress or periods of profound salt depletion^117–120^. It is also likely that APMs retain sensitivity to potassium, with aldosterone release providing essential protection against hyperkalaemia. However, studies of laser-dissected APMs have found *CACNA1D* mutations in 50–60%, as well as mutations in other genes linked to aldosterone production^17,38,107^. The *CACNA1D* mutations found in APMs are not always the same as those found in the adjacent APA^17^. However, transition of an APM into an APA has been captured in adrenal sections^121,122^, and it is likely that at least some APMs are a pathological precursor of mutant *CACNA1D*-containing APAs.

Unlike transcriptome analyses of APAs, which can be readily performed owing to their abundance, whole-transcriptome analyses of APMs are constrained by the technical challenge of extracting small amounts of RNA from laser-based microscopic sample dissection and collection^107,123^. A 2015 microarray analysis of RNA from APMs and three normal adrenocortical zones corroborated or anticipated many of the genes upregulated in APAs that were not found in the non-CYP11B2-expressing ZG^107,124^. For example, *MC2R*, which encodes the ACTH receptor, and *CLRN1*, which has been linked to hearing and vision, were upregulated in the APM samples and are upregulated in most APAs. Other genes, including *DFNB31* (encoding WHRN), which, like *CLRN1*, has been linked to deafness^125–127^, are also increased in APAs, especially in ZG-like APAs. The mechanisms that underlie hearing and sight in the inner ear and retina, respectively, may have some commonalities with those involved in aldosterone regulation. For example, *CACNA1D* is expressed in the retina and inner hair cells of the ear^128^, and *Cαcnα1d*-deficient mice and humans with homozygous loss-of-function, are deaf^129^. Another group of genes, including *NPHP1* and *CFAP221*, that are upregulated in APMs, are associated with primary cilia function, suggesting a possible role in sensing of the ionic environment^130^. Many of the genes upregulated in APMs compared with the ZG are also abundant in the ZF and/or zona reticularis (ZR), inferring that it is their down-regulation in ZG that is notable, and presumably related to the remarkable suppression of aldosterone synthase in the majority of the adult human ZG. Evidence that we should indeed focus as much on the genes that are down-regulated in ZG adjacent to an APA (presumably due to negative feedback from aldosterone and/or Na^+^ excess) as on the genes upregulated in APMs, comes from our microarray analysis in which we compared 14 pairs of ZG and ZF samples adjacent to an APA with 7 pairs of ZG and ZF samples adjacent to a phaeochromocytoma. Although the latter pairs of adrenal samples are an imperfect ‘normal control’, phaeochromocytoma has the merit of causing Na^+^ loss (through pressure natriuresis)^131^. Several ‘APM-associated genes’, including *CYP11B2* itself, as well as the ACTH receptor gene *MC2R*, the neuronal genes *NETO2* and BEX1, and *TSPAN12*, had 2–8-fold lower expression in ZG samples adjacent to an APA than in ZG samples adjacent to a phaeochromocytoma, for example_20,107,115,132–139_.

Our 2015 microarray study also identified several genes that were upregulated in APMs (compared with their expression in the three physiological adrenocortical zones), but were not previously associated with aldosterone regulation. For example, *SLC35F1* encodes a neuronal decamembrane-spanning protein from the SLC35F family of nucleotide sugar transporters. *TMEM266* is one of several genes that is restricted to the ZG and cerebellum^140^. Its ability to undergo rapid structural changes (that is, within milliseconds) in response to voltage changes could explain the pacemaker activity of ZG cells^141–144^. The expression of *TMEM266* in APMs, and *SLC30A1* in APAs (discussed earlier), shed light on roles of zinc in aldosterone regulation. Coincidentally, *CASZ1* encodes a zinc finger transcription factor that may function as a tumour suppressor. It was one of only two positive loci in a large genome-wide association study of PA, providing evidence that at least some APMs are causally linked to PA^145^.

One caveat to the interpretation of findings from transcriptome analyses of APMs is the frequent need to pool RNA from several APMs, given their small size. This limitation has made it difficult to investigate heterogeneity in an adrenal – for example, to assess whether some APMs function physiologically as an emergency reserve of aldosterone production and others represent a pre-tumorous pathology. Developments in single-cell technology enabling in situ analyses, may resolve such questions. However, findings from one study that used in situ metabolomics indicates that two types of APM are likely to exist, one of which demonstrates similarity to APAs^117^.

### Translation of molecular pathogenesis

The discovery, in APAs, of recurrent somatic mutations in ion channels and transporters was consistent with earlier studies of adrenocortical cell function and rodent models of PA. Functional studies of adrenocortical cells had shown that the hyperpolarization of ZG cells explained their exquisite sensitivity to elevations of extracellular K^+^, whereas knockout models of K^+^-channels demonstrated the potential for depolarization to cause PA^146,147^. Paradoxically, this consistency with prior studies limited the initial impact of the molecular findings, and their potential for clinical translation^29^. However, three key insights from studies published since 2020 may have changed this trajectory, leading to the validation of non-invasive molecular probes as an alternative to AVS; demonstrating translatable correlations of somatic genotype with clinical presentation and outcomes following adrenalectomy; and yielding proof-of-concept in a prospective evaluation of a minimally invasive alternative to adrenalectomy.

### Molecular imaging of APAs

The MATCH study was a prospective, randomized controlled study that used a within-patient design to compare the accuracy of a non-invasive test – 11C-metomidate PET CT (MTO) – with AVS in predicting the outcome from adrenalectomy in patients with PA^63^. Complete or partial biochemical success was achieved in 93% of the 78 patients predicted by MTO and/or AVS to have unilateral PA. Statistically, MTO was non-inferior to AVS for predicting biochemical and clinical success following adrenalectomy. However, its requirement for on-site synthesis, and for dexamethasone pre-treatment, means that MTO is unlikely to remain the PET ligand of choice, and will be replaced by longer-acting, and/or more CYP11B2-selective ligands^148–152^. Nevertheless, MATCH established the principle of molecular imaging as a non-invasive, operator-independent alternative to AVS.

#### Somatic genotype explains and predicts clinical outcome

The MATCH trial also identified correlations between somatic genotype and clinical presentation and outcomes following adrenalectomy. Logistic regression of the effect of age, gender, blood pressure and APA genotype on surgical outcome found that APA genotype was the strongest predictor. 14 of 18 patients with *KCNJ5* mutations, compared with 1 of 20 patients with *CACNA1D* mutations, achieved complete success, which was sustained for at least 2 years^63^. Although somatic genotype was a post-operative finding, *KCNJ5* mutations correlated strongly with pre-operative measurements of urinary hybrid steroids (that is, the 18-hydroxy cortisol-to-cortisol ratio) (Fig. 3).

Circumstantial evidence suggests that *CACNA1D* mutations are frequently bilateral^38^. Not only are they the most common mutation in APMs, but they probably also underlie the pathology of bilateral ‘hyperplasia’. This proposal is supported by the finding in one study that 15 of 15 adrenals with bilateral hyperplasia had APMs, compared with 4 of 15 with diffuse CYP11B2 positivity; of 99 APMs isolated from these adrenals, 57 (58%) had mutations in *CACNA1D* whereas only one had a mutation in *KCNJ5* (ref. 38). Of note, the same adrenal gland may have up to eight different *CACNA1D* mutations in different APMs^17^. The multiplicity of spontaneous *CACNA1D* mutations, and their finding in the adrenals of patients with proven bilateral disease, suggests that mutations in this gene commonly occur in both adrenals. The converse is true of *KCNJ5* mutations, which may explain why *KCNJ5*-mutant tumours are usually solitary, and hypertension is usually cured by their removal. Moreover, *CACNA1D* mutations are also more common than *KCNJ5* mutations in Black patients^60,63^; in the MATCH study, it was the self-identified Black patients with *CACNA1D* mutations who were least likely to achieve either biochemical or clinical success.

Candidate surrogates for *CACNA1D* mutations, which would have the potential to identify patients, likely to achieve as much benefit from medical as surgical therapy, have been suggested by transcriptome analyses of APAs^88^. These studies have identified neuronal proteins, such as neurexins, as potential candidates, because their RNA expression was highest in *CACNA1D*-mutant samples. The neurexins are cleaved into soluble ectodomains, which are anticipated to have low background levels because of the blood–brain barrier^153^.

#### Molecular imaging guides minimally invasive alternative to surgery

Although molecular imaging was developed to replace invasive AVS, it also has a potential role in facilitating alternatives to surgery. Radiofrequency ablation may provide an alternative to adrenalectomy, particularly for small adenomas; however, studies to date, mainly by the percutaneous route, suggest that a trade-off exists between safety and efficacy, as the electrical field from the centre of a non-spherical adenoma may encroach on adjacent adrenal medulla, stimulating adrenaline release and a peri-procedural pressor crisis^154–157^. Given the proximity of the left adrenal gland to the stomach, we have performed a proof-of-concept study of endoscopic ultrasound-guided transgastric radiofrequency ablation of PET-imaged APAs in 28 patients. The adenoma, in this approach, is both seen and approached from the stomach, and ablation is achieved by multiple short bursts as the catheter is continually re-positioned. Preliminary findings^158^ suggest that this approach is feasible and safe. Both percutaneous and endoscopic approaches need to be formally compared, with each other and with adrenalectomy, and a prospective RCT is now in progress^159^.

### Therapeutic implications

Existing calcium-channel blockers (CCBs) are non-selective and block both the vascular Ca_v_1.2 and 75% identical Ca_v_1.3, which is expressed mainly in parts of the nervous system (such as the cerebellum and inner ear cells) and endocrine organs. CCBs probably reduce aldosterone secretion in PA, and their use is often regarded as a ‘confounding medicine’ to be withdrawn during diagnostic tests. However, evidence of their ability to reduce autonomous aldosterone secretion is anecdotal^160^, and even less is known about the efficacy of CCBs to reduce aldosterone secretion by *CACNA1D*-mutant compared with other APAs. In mice, heterozygous deletion of *Cyp11b2* does not induce a phenotype, suggesting that a >50% reduction in aldosterone secretion is required for a clinical effect^161^. The dose of CCBs is effectively limited by their Cav1.2 (vascular) effects, particularly peripheral oedema; hence, a selective Ca_v_1.3 inhibitor used at maximal dose is in theory attractive. In studies of an adrenal cell line transfected with two separate, electrophysiologically distinct *CACNA1D* mutants, excess aldosterone secretion consequent upon augmented calcium influx was inhibited by a selective antagonist of Ca_v_1.3, 1-(3-chlorophenethyl)–3-cyclopentylpyrimidine-2,4,6-(1H,3H,5H)-trione^162^, and by the maximum soluble concentration of a non-selective antagonist of L-type calcium channels, dihydropyridine calcium channel antagonist, nifedipine^163^. Targeting of Ca_v_1.3 may have particular value in two common groups of patients in whom *CACNA1D* mutations are suspected to predominate. As discussed earlier, these are patients of African ancestry, even if investigations suggest unilateral disease, and any patient in whom investigations have suggested bilateral disease^17,38,107^.

Loss of potassium selectivity in the two most common *KCNJ5* mutants combined with a loss of sensitivity to the potassium channel blockers barium and tertiapin-Q led to the suggestion that molecules could potentially be identified that block ion passage through the mutant, but not the wild type, channel. Indeed, in an adrenal cell line expressing the mutant *KCNJ5*, the macrolide antibiotic roxithromycin markedly inhibited *CYP11B2* expression and aldosterone production^164^. Similar effects were observed with idremcinal, a macrolide motilin receptor agonist, and with synthesized macrolide derivatives that lacked antibiotic or motilide activity. Macrolide-derived selective inhibitors of mutant *KCNJ5* thus have the potential to advance the diagnosis and treatment of *KCNJ5*-mutant APAs^164^.

### Future perspectives

The finding that the ontogeny of adrenal glands in primates is fundamentally different from that of rodents should prompt the exploration of alternative or additional explanations for the physiological and pathological regulation of aldosterone^165^. The current centripetal migration model of adrenocortical development describes a common origin for gonadal and adrenocortical cells, with the latter constantly renewed from stem cells in the outer adrenal capsule, which metamorphose sequentially into ZG, ZF and ZR cells^166^. However, new research has demonstrated that primate adrenal tissue does not originate from an adrenogenital ridge^165^. Testing whether centripetal migration is the correct model for human adrenal gland development is difficult but becoming feasible through the application of single-cell analyses, which use somatic DNA variants as barcodes for cell ancestry^167,168^. Questions about cell ontogeny in human adrenal are raised by the finding of juxta-medullary ZG cells, by the presence of adrenomedullary cells in the ZG^169,170^ and by our finding that RSPO3-LGR5 signalling inhibits aldosterone production in the human adrenal gland^105,169,170^. This observation seems contrary to the role of RSPO3 in rodent adrenal, where it stimulates replenishment of ZG cells that have migrated centripetally^110^.

The distinctive clinical and molecular phenotypes of the APAs with *GNA11, GNAQ* or *CADM1* mutations suggest that further insights might be gained from the identification of additional genes that are recurrently mutated, especially if they cause presentations of PA that are currently overlooked. Although the 2023 discoveries of *CADM1* and *SLC30A1* mutations indicate that recurrent somatic variants of unexpected genes may still await discovery, realistically, most new mutations are likely now to be private – meaning that they are rare and occur in single families or patients. As experiments of nature, we can still learn from them, but it will be difficult to be confident that they are causally related to PA in the absence of functional studies. We have identified one such somatic mutation in *DPYSL2* (also known as *CRMP2*) – this gene is enriched in the ZG, is associated with ion-channel trafficking and is likely to bind to the β-subunit binding site of Ca_v_1.3^63,171,172^. Another mutation was identified in *VAPA*, which has also been linked to protein trafficking^63,173^ (Fig. 4). Moreover, the most upregulated gene in double-mutant APAs (that is, APAs with a double mutation in *GNA11* or *GNAQ* and *CTNNB1)*, the cerebellar gene *TMEM132E*, also has a role in trafficking a family of cholinergic receptors that are ion channels^89,174^. These findings suggest that we should investigate accessory proteins – such as those involved in channel trafficking – for their pathogenic role in disease onset. If we think of the molecular causes of PA as the potential targets of curative therapy, then such accessory proteins may provide as many opportunities for therapeutic intervention as the aldosterone synthetic pathway itself.

**Fig. 6:**
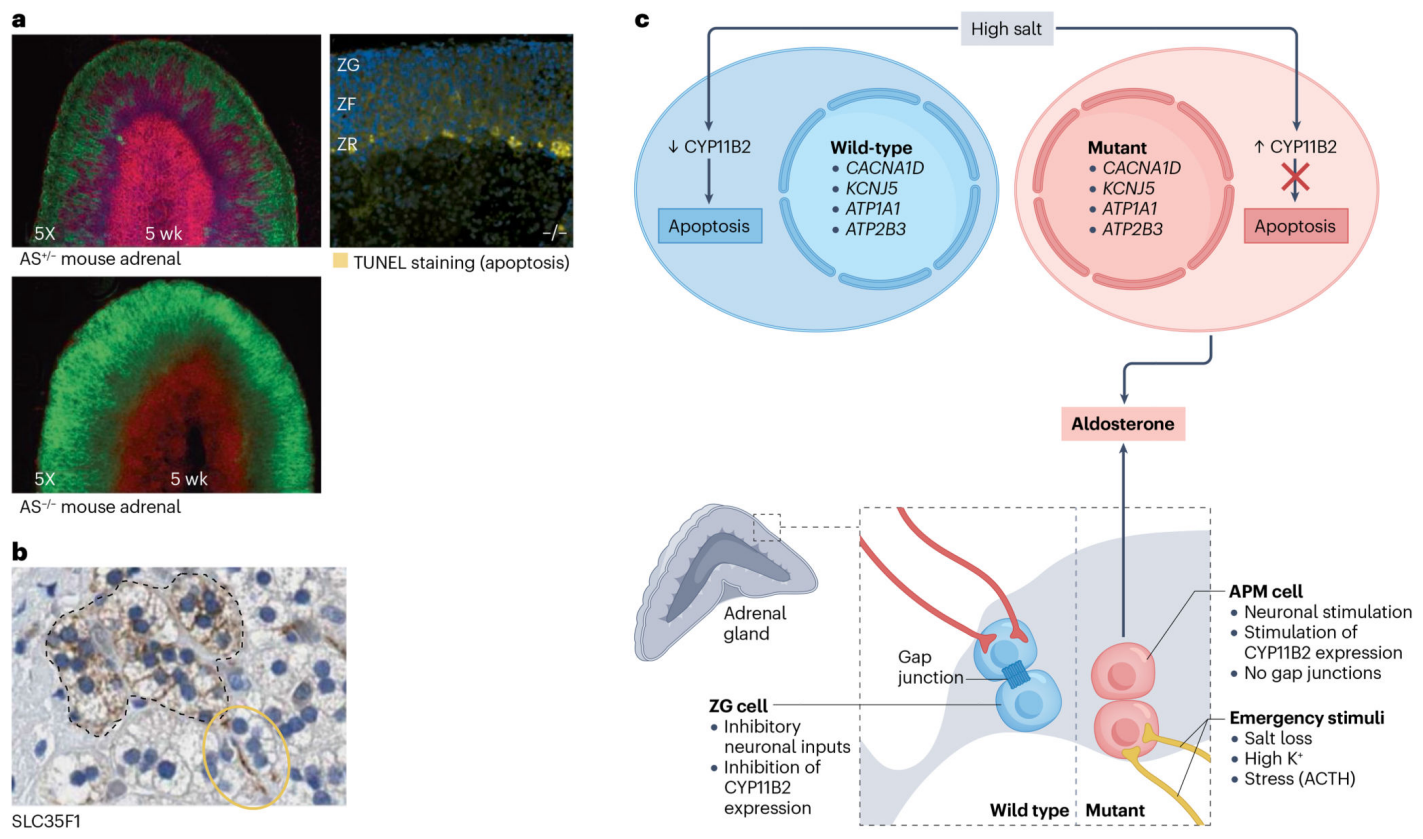
A proposal for the evolutionary selection of aldosterone-producing adenomas and aldosterone-producing micronodules. **a**, Confocal analysis of adrenal tissue from mice with heterozygous (A^+/−^) and homozygous (AS^−/−^) knockout of the gene that encodes aldosterone synthase (*CYP11B2*) shows that homozygous *CYP11B2* deletion induces centripetal migration of outer zona glomerulosa (ZG; green) cells towards the inner zona fasciculata (ZF) layer by 5 weeks of age^166^. At 6 months, ZG cells that have migrated to the zona reticularis (ZR)–medulla boundary in AS^−/−^ mice expresses markers for apoptosis, terminal deoxynucleotidyl transferase dUTP nick end labelling (TUNEL) staining (yellow)^161^. **b**, The neuronal gene, *SLC35F1* is highly expressed both in APM cells (encapsulated by the dashed brown lines) and in nerve-like structures adjacent to these (yellow circle)^195^. **c**, We propose that salt-induced suppression of aldosterone synthesis in the ZG is a scenario analogous to that induced by genetic *CYP11B2* deletion. We also propose that the presence of mutations in aldosterone-driver genes such as *CACNA1D* may facilitate the evolutionary selection of APMs, by imparting a survival advantage. The presence of gap junctions between normal cells of the ZG may enable propagation of neuronal signals and thereby also contribute to the inhibition of aldosterone production in this region. By contrast, the main gapjunction proteins are largely absent from APMs. We also propose that neuronal excitation may over-ride negative feedback regulation in APMs and contribute to autonomous CYP11B2 production. Note that this model in which APMs arise from clonal selection is unproven. Part **a** adapted from ref. 161, Oxford Academic Press, and ref. 166, Elsevier. Part **b** is from The Human Protein Atlas^196^, image available at https://www.proteinatlas.org/ENSG00000196376-SLC35F1/tissue/adrenal+gland#img.

Of greater interest now than the search for individual new mutations is the question of why functional somatic mutations in the adrenal cortex are overall so common, and whether a therapy that reverses the consequence of these mutations would cause an involution of the APAs. Since our first report of somatic mutations a decade ago^22^, we have speculated that the driver is the reported apoptosis of CYP11B2- expressing ZG cells when *CYP11B2* itself is inhibited or genetically deleted^120,161,166^. The complete suppression of aldosterone synthase expression in most adult ZG cells outside the APMs – which we attribute to excess salt intake – is associated with the appearance of apoptotic cells within the ZG itself, rather than at the ZR–medullary boundary^105^ (Fig. 6). An inference from these observations is that a therapy that suppresses aldosterone synthesis may cure PA. Therapeutically, the most direct route to switching off aldosterone production is inhibition of aldosterone synthase. After many years during which candidate drugs demonstrated insufficient selectivity to avoid inhibiting 11β-hydroxylase – which is responsible for cortisol synthesis and exhibits close homology to aldosterone synthase – a 12-week phase II trial of baxdrostat, which exhibits 100:1 selectivity for aldosterone synthase, demonstrated a comparable blood pressure reduction in patients with resistant hypertension to that achieved previously in similar patients using spironolactone^120,175^.

The long-term effects of baxdrostat, both in hypertension and in PA, will be of interest. Selective inhibition of Ca_v_1.3 may also hold promise. Although no such selective inhibitor currently exists, structural modelling has identified that the antihistamine, cinnarizine, can bind and block the central pore cavity of Ca_v_1.3^176^.

There are obvious public health implications if the change in aldosterone synthase expression, from diffuse to almost absent, between children and adults is salt-induced. We do not yet know whether salt is the sole culprit or whether suppression of renin–angiotensin is the sole mediator of aldosterone suppression. The renin status of patients at the time of adrenalectomy is not usually published. However, our prospective comparison of surgically and medically treated patients, in which all patients were force-titrated to spironolactone 100 mg or a maximum tolerated equivalent, showed that this dose lifted the suppression of renin in most patients^63^. These findings support our suspicion that withdrawal of ANGII (the downstream product of renin secretion) is not the sole factor in the disappearance of CYP11B2 from most adult human adrenals. Moreover, many of the genes that are upregulated in the ZG region in humans, such as *LGR5*, inhibit aldosterone production, contrary to the expectation for prominent genes in an endocrine gland, and cause apoptosis of transfected adrenocortical cells^105^. In addition, the presence of gap junctions between cells of the ZG may enable propagation of neuronal signals and thereby also contribute to the inhibition of aldosterone production in this region^95^.

*LGR5* and *GJA1* (the main gap junction protein) are largely absent from APMs, along with the ZG-specific transcripts for the inhibitory GABA receptor B (*GABBR2*) and neuronal cadherin (*CDH12*). What is unknown, however, is whether APMs are a mutation-driven, pre-tumorous pathology or whether there is a key cell or process (for example, an excitatory and inhibitory neuronal input), that is extrinsic to APMs and non-aldosterone producing ZG-glandular cells, and determines the delineation between these. The most up-regulated gene in APMs, after CYP11B2 itself, is a neuronal gene, *SLC35F1*, that is highly expressed both in APM cells and in nerves innervating these (Fig. 6). Indeed, several of the most upregulated transcripts in APMs and/or APAs are either neuronal (*PCP4, KIAA1549L, KCND3, NRXN2, PPP1R17, SV2B*), with highest expression typically in the cerebellum, or neuroendocrine (UNC79, CADM1, VAT1L), with high expression in the adrenal medulla and pituitary^20,63,107^. Most of the ‘neuronal genes’ are enriched in APAs of particular genotypes, namely *CACNA1D-, ATP1A1*- and *ATP2B3*-mutant APAs, but are virtually absent from *KCNJ5*- and double-mutant APAs^63^. Whether individual neurotransmitters are exciting different cells-of-origin for each APA genotype and thereby over-riding negative feedback regulation or whether cerebellar-type synapses between ZG cells are critical to the oscillation of currents around the glomeruli is unknown, but may soon be addressed by the emergence of new techniques for in situ transcriptomic analyses.

Neuronal input does not necessarily explain the ectopic expression of neuronal genes. An additional clue to the mechanisms underlying ZG pathophysiology may be the high abundance in ZG and certain APAs of three molecules – *VPREB3, SHOC1* and *KIAA1210* – that have no obvious reason to be expressed in adrenal tissue. The only known function of *VPREB3* is in pro-B lymphocytes, where its encoded protein acts as part of a proxy B cell receptor for testing permutations of recombined immunoglobulin genes; however, it is also richly expressed in cerebellar Purkinje cells^177^. SHOC1 functions in meiosis, by promoting and protecting sites of DNA recombination; it is also highly expressed in the XY body^178^. *KIAA1210*, also has a specific function in gonads, being found in cell bridges between spermatogonia and (like SHOC1) the XY body. KIAA1210 regulates the dynamic changes in chromatin structures that occur during spermiogenesis^179,180^. Microarray studies and the Human Protein Atlas show high ZG and capsular expression of these three genes^64^. One suggestion is that the expression of all three molecules might be invoked in adrenal tissue by cell fusion and its sequelae. Such cell fusion is used by various cell types, including trophoblasts, myoblasts, osteoclasts, hepatocytes (in animal studies) and cancer cells^181–184^, as an alternative to stem cell differentiation for the replenishment of tissue^181,182,185,186^. Evidence of cell fusion within adrenal tissue is suggested by the finding of occasional hybrid adrenocortical and adrenomedullary cells on electron microscopy, and should be readily confirmed by single-cell sequencing analyses^187^. Cell fusion would not only allow ZG cells to synapse with each other but may also offer an opportunity for a form of meiotic recombination to occur during the subsequent ploidy reduction and establishment of synkaryons. These hybrid cells, with restored diploidy, would likely reproduce normally and transcribe two copies of each gene. However, their fitness to regulate and produce essential adrenal steroids may be increased by the merger of two types of ‘cell machinery’ and by selection of cells with two copies of the fittest parental gene (rather than one of each). The high adrenal expression of *APOBEC3C*, a single-strand DNA editing gene associated with hypermutation in immune cells, and *UNG*, a DNA-repair gene, indicate that, as in immune cells, gene ‘improvement’ may not be limited to shuffling (recombination) but may also involve somatic mutation. Selection of ‘improved’ genes arises, we propose, through the need of adrenocortical cells to survive oxidative steroidogenesis, migration and transformation, which successively generates two essential steroids. However, under conditions of salt excess, ZG steroidogenesis becomes a futile release of reactive oxygen species, as no steroid precursor is offered as a co-substrate with molecular oxygen to the steroidogenic electron transport chain^188,189^. Under such circumstances, the process of mutation and recombination promotes, we propose, the emergence of cells that constitutively produce aldosterone, restoring oxygen usage, and protecting against the apoptosis seen in models of aldosterone synthase deletion^161,166^.

## Conclusions

Earlier we mentioned peptic ulcers as an historical analogy and challenge. Two obvious differences with the stomach may explain why adrenal pathology has received far less attention. One is the relative sizes of the adrenal gland and stomach, and the other is that we have two of the former. These anatomical differences have dulled the need to find treatments less brutal than the removal of one of the adrenal glands, while justifying the use of complex tests to answer a question that may be misleading – that is, whether the PA is unilateral or bilateral. In the MATCH trial, two thirds of patients had unilateral disease, which improved after surgery, but only one third of these patients achieved a sustained, complete cure. Shifting priorities to the 99% of patients with undiagnosed PA presents two clear questions: first, how to identify patients who have a high likelihood of complete cure following non-pharmacological intervention, and second, how can PA be cured in the remainder.

A growing body of literature suggests that distinct genotype–phenotype patterns and clinical surrogates for genotype will facilitate diagnosis and enable prediction of individual patient outcomes. The hope is that at least one of the gene mutations, proteins, cells or pathways discovered will be sufficiently unique to autonomous aldosterone production that it is targeted to switch off autonomous aldosterone production through the delivery of a small drug, RNA or gene-editor. Notably, the adrenal cortex, in its hunger for cholesterol as precursor to steroidogenesis, has a far higher density than any other tissue of receptors for LDL and HDL. Lipid nanoparticles incorporate one of these lipids to deliver RNA-based therapies; and receptors for both lipids are enriched in cells with autonomous aldosterone production. Suicide therapy surely beckons.

## Supplementary Material

Supplementary Material

## Figures and Tables

**Table 1:** Mutations associated with primary aldosteronism

Gene	Occurrence	Mechanism of action	Refs.
*ATP1A1*	Somatic in APAs	Impaired pump function and pathologically channel like-permeabilities for Na^+^, H^+^ and Ca^2+^	22,67
*ATP2B3*	Somatic in APAs	Channel like-permeabilities for Na^+^, H^+^ and Ca^2+^	67
*CACNA1D*	Germline in PASNA and somatic in APAs	Increases Ca^2+^ permeability	22,75
*CACNA1H*	Germline in FH-IV and somatic in APAs	Increases Ca^2+^ permeability	81,82
*CADM1*	Somatic in APAs	Inhibition of gap junctions and modulation of biological rhythms	95
*CLCN2*	Germline in FH-II and somatic in APAs	Increases Cl^-^ efflux	85
*CTNNB1*	Somatic in APAs and adrenocortical carcinomas	Prevents β-catenin degradation, which increases signalling via the TCF/LEF family	190
CYP11B1/2 hybrid gene	Germline in FH-I	Pathologically activated by ACTH via MC2R and cAMP signalling	40
*GNA11* and *GNAQ*	Somatic in APAs	Prevents termination of G protein signalling downstream of the AT1R, leading to increased calcium release from intracellular stores Co-occurs with *CTNNB1* mutations	89
*KCNJ5*	Germline in FH-III and somatic in APAs	Abnormal Na^+^ influx	18
*SLC30A1*	Somatic in APAs	Increases Zn^2+^ permeability	100

Many of the mutations cause membrane depolarization, activation of voltage-gated calcium channels, calcium influx and increased calcium signalling, which stimulates CYP11B2 expression and aldosterone production. ACTH, adrenocorticotropic hormone; APAs, aldosterone-producing adenomas; AT1R, angiotensin II type 1 receptor; FH, familial hyperaldosteronism; MC2R, melanocortin 2 receptor; PASNA, primary aldosteronism with seizures and neurological abnormalities; TCF/LEF, T cell factor/lymphoid enhancer factor.

## Box 1

Key findings from studies of somatic mutations in APAs

- 80-95% of aldosterone-producing adenomas (APAs) have a somatic mutation within key regions of the transporters encoded by *KCNJ5, ATP1A1* and *ATP2B3*, or near the Ca^2+^ pores of the calcium channel encoded by *CACNA1D*^17,63^.
- The highest numbers of mutations have been identified in CYP11B2-positive regions of APAs, representing the areas that express aldosterone synthase^17^.
- In adult human adrenals, CYP11B2 is largely confined to microscopic clusters, termed aldosterone-producing micronodules; a mutated aldosterone-driver gene can be found in 30-60% of these clusters, mainly involving *CACNA1D*^36^.
- The phenotype of *KCNJ5*-mutant APAs differs from that of other APAs: clinically, *KCNJ5*-mutant APAs present more frequently in younger individuals and women; macroscopically, they are larger; and microscopically, they comprise large, foamy cells; they also differ in their gene expression (demonstrating co-expression of zona fasciculata (ZF) enzymes, *CYP11B1* and *CYP17A1*)^21,58,63^.
- APAs with mutations in *CACNA1D, ATP1A1* or *ATP2B3* are more similar to each other than to *KCNJ5*-mutant APAs, and more similar than *KCNJ5*-mutant APAs to the microscopic aldosterone-producing micronodules^22,63,106,107^.

The translational questions arising from these findings are:

- Whether clinical outcomes or management should differ between genotypes, and if so, whether these differences can be predicted prior to surgical removal of the APA?
- Whether the high prevalence of APAs is explained by their pathogenesis and links between this and mechanisms of aldosterone regulation in adult humans?

Answers to these questions may come from our understanding of the ZF phenotype of *KCNJ5*-mutant APAs, and the inferred co-existence of *CYP11B1* and *CYP11B2* (encoding 11-β hydroxylase and aldosterone synthase) within the same cell^197^. The consequences of coexistent *CYP11B1* and *CYP11B2* can be inferred from studies of patients with familial glucocorticoid remediable aldosteronism (GRA). GRA is caused by a rare germline chimaera between the two genes, which results in production of the hybrid steroids 18-hydroxycortisol and 18-oxocortisol, as has also been shown for patients with *KCNJ5* mutations^41,197,198^. Steroid profiling using liquid chromatography with tandem mass spectrometry (LC-MS/MS) has enabled measurement of these hybrid steroids^199^ and have demonstrated levels of the hybrid steroids to be higher in APAs than in bilateral adrenal hyperplasia^200,201^. Steroid profiling of peripheral blood using LC-MS/MS has potential value particularly for the identification of *KCNJ5*-mutant APAs, and may enable the identification of candidates for whom invasive adrenal vein sampling (AVS) can be avoided and substituted with findings of solitary adenoma on CT or MRI, or in whom complete remission of hypertension post-adrenalectomy is most likely^29,202^. However, steroid profiling using LS-MS/MS requires expensive instrumentation and a high level of expertise. More widely available at present is the overnight dexamethasone suppression test based on the often-overlooked finding that dexamethasone failed to suppress either cortisol or aldosterone in siblings first described to have germline *KCNJ5* mutations^41^.

## Key points

- Primary aldosteronism (PA) is a common cause of hypertension; it is potentially curable when caused by a unilateral aldosterone-producing adenoma (APA), but it can lead to resistant hypertension and a high cardiovascular risk in cases in which PA is undiagnosed.
- Current clinical guidelines and practice centres on risky tests and procedures, such as saline suppression, adrenal sampling and surgery, and poorly tolerated drugs such as spironolactone; these practices have remained largely unchanged over the past 30 years.
- By contrast, the discovery of somatic mutations over the past decade has identified these as the cause of autonomous aldosterone production in most patients with PA, including 80–90% of APAs; the identified mutations most commonly affect the ion-channel genes *KCNJ5* and *CACNA1D*.
- Several distinct genotype–phenotype patterns and emerging clinical surrogates for genotype suggest the potential for molecular discovery to predict individual patient outcomes, permitting rational stratification of interventions.
- In vivo molecular ligands for aldosterone synthase have been validated for non-invasive PET CT detection of APAs; on the left side, ultrasound-guided endoscopic radiofrequency ablation of PET-imaged APAs seems to be a safe alternative to surgery.
- Genetic deletion models suggest curative potential for the suppression, rather than blockade, of aldosterone synthesis.
