# Supplementary material for: Primary aldosteronism: molecular medicine meets public health
Source: Nat Rev Nephrol. Author manuscript; Available in PMC 2023 Dec 1. (PMC7615304; doi:10.1038/s41581-023-00753-6)
Supplement: Supplementary Material [file EMS187489-supplement-Supplementary_Material.pdf]

Supplementary Table 1 | 40 genes with at least 4-fold higher expression in ZG than ZF

Gene Symbol	Fold-Change (G vs. F)	p-value	Gene Name
LGR5	25.0	1.6E-23	leucine-rich repeat-containing G protein-coupled receptor 5
VSNL1	23.6	3.6E-23	visinin-like 1
ANO4	19.9	6.6E-24	anoctamin 4
NEFM	14.8	9.2E-12	neurofilament, medium polypeptide
VCAN	14.3	1.9E-13	versican
DACH1	14.2	1.4E-21	dachshund homolog 1 (Drosophila)
NR4A2	10.9	6.2E-08	nuclear receptor subfamily 4, group A, member 2
KIAA1210	8.9	5.4E-11	KIAA1210
SFRP4	8.7	2.3E-10	secreted frizzled-related protein 4
C12orf75	7.2	4.4E-13	chromosome 12 open reading frame 75
C9orf84	7.0	5.1E-13	chromosome 9 open reading frame 84
VAT1L	6.9	9.2E-17	vesicle amine transport protein 1 homolog
EDNRA	6.5	1.2E-16	endothelin receptor type A
OGN	6.3	5.2E-12	osteoglycin
NR4A3	5.9	3.1E-08	nuclear receptor subfamily 4, group A, member 3
SESN3	5.9	6.1E-16	sestrin 3
LEF1	5.9	4.3E-15	lymphoid enhancer-binding factor 1
PLD5	5.9	2.6E-12	phospholipase D family, member 5
CDH12	5.8	4.5E-10	cadherin 12, type 2 (N-cadherin 2)
HLF	5.7	5.7E-16	hepatic leukemia factor
TMEM176A	5.6	1.1E-08	transmembrane protein 176A
LAMP5	5.6	7.1E-11	lysosomal-associated membrane protein family, member 5
PDE5A	5.6	2.2E-15	phosphodiesterase 5A, cGMP-specific
GABBR2	5.4	8.3E-13	gamma-aminobutyric acid (GABA) B receptor, 2
PTGDS	5.4	1.1E-14	prostaglandin D2 synthase 21kDa (brain)
POSTN	5.4	7.5E-08	periostin, osteoblast specific factor
RSPO3	5.3	3.3E-11	R-spondin 3 homolog (Xenopus laevis)
RBP4	5.0	3.8E-07	retinol binding protein 4, plasma
EGR3	5.0	9.9E-09	early growth response 3
PDE2A	4.7	1.3E-16	phosphodiesterase 2A, cGMP-stimulated
COL14A1	4.7	3.1E-15	collagen, type XIV, alpha 1
BICC1	4.7	5.3E-11	BICC1
LMOD1	4.5	3.0E-11	leiomodin 1 (smooth muscle)
CACNB2	4.5	2.6E-10	calcium channel, voltage-dependent, beta 2 subunit
PCP4	4.4	5.7E-06	Purkinje cell protein 4
ATP10A	4.4	5.3E-15	ATPase, class V, type 10A
TIPARP	4.3	8.7E-05	TCDD-inducible poly(ADP-ribose) polymerase
MFAP5	4.1	1.1E-06	microfibrillar associated protein 5
NR4A1	4.1	2.5E-07	nuclear receptor subfamily 4, group A, member 1

*Published in Table S3 of reference [1]

Reference

1. Zhou, J., et al., *DACH1, a zona glomerulosa selective gene in the human adrenal, activates transforming growth factor-β signaling and suppresses aldosterone secretion.* Hypertension, 2015. **65**(5): p. 1103-10.
